# Growth Enhancement of *Salmonella* by Tungstate Treatment

**DOI:** 10.3390/pathogens15050478

**Published:** 2026-04-29

**Authors:** Robin C. Anderson, Delila D. Dominguez, Megan R. Shaw, Casey N. Johnson, Samat Amat, Jackie M. Kotzur, Merritt L. Drewery, Patricia J. Baynham, Ken J. Genovese, Tawni L. Crippen, Ryan J. Arsenault

**Affiliations:** 1 Food and Feed Safety Research Unit, Southern Plains Agricultural Research Center, United States Department of Agriculture/Agricultural Research Service, College Station, TX 77845, USA; casey.n.johnson@usda.gov (C.N.J.); jackie.kotzur@usda.gov (J.M.K.); ken.genovese@usda.gov (K.J.G.); tc.crippen@usda.gov (T.L.C.); ryan.arsenault@usda.gov (R.J.A.); 2 Department of Agricultural Sciences, Texas State University, San Marcos, TX 78666, USA; ddd921@nmsu.edu (D.D.D.); m_d553@txstate.edu (M.L.D.); 3 Department of Biological Sciences, St. Edward’s University, Austin, TX 78704, USA; mshaw2@stedwards.edu (M.R.S.); patricib@stedwards.edu (P.J.B.); 4 Department of Animal Sciences, North Dakota State University, Fargo, ND 58108, USA; samat.amat@ndsu.edu

**Keywords:** foodborne pathogen, nitrate, nitrite, ruminal microbiota, *Salmonella*, tungstate

## Abstract

*Salmonella* in gut habitats have traditionally been thought to conserve energy for growth via fermentation. However, recent reports indicate that ingested *Salmonella* can stimulate host-derived nitrate accumulation in the mucosal microenvironment, thereby enabling growth through nitrate respiration. Sodium tungstate is an effective treatment that inhibits the growth of certain nitrate-respiring bacteria, including *Escherichia coli, Paracoccus* and *Proteus*, when cultured under gut simulating conditions or within the gut of experimentally treated mice. This inhibitory effect is hypothesized to occur by inactivation of molybdenum-containing enzymes required for nitrate metabolism. Information is lacking on whether tungstate can inhibit the growth of *Salmonella*, particularly in the presence of culturable gut microbiota. Therefore, the objectives of this study were to evaluate the effects of sodium tungstate on *Salmonella* during pure culture or when cultured with freshly collected bovine rumen microbiota and to assess its impact on fermentation as well as nitrate and nitrite metabolism within the rumen microbial cultures. Our results indicate that 50 mM sodium tungstate treatment, whether alone or in combination with 5 mM nitrate, markedly increased the growth of *Salmonella* serovars Newport, Dublin and Typhimurium during pure culture. Moreover, during in vitro incubation, increased growth of experimentally inoculated *S.* Newport as well as wildtype *E. coli* and lactic acid bacteria was observed with ruminal microbiota treated with 100 mM tungstate when compared to non-tungstate-treated controls. Effects of tungstate on nitrate and nitrite metabolism were as expected during pure and mixed culture. When cultured with reduced tungsten rather than tungstate, the latter being bound to four oxygen atoms, an inhibitory effect on the growth of *S*. Newport was observed and effects on nitrate and nitrite metabolism were consistent with those observed with tungstate. These results suggest that, under conditions used in the present experiments, tungstate may have served as a source of oxygen for respiration above that achieved with nitrate alone. While this hypothesis has yet to be proven, it is supported by an adverse effect of tungstate, whether alone or in combination with 5 mM nitrate, on methane and volatile fatty acid production by the ruminal microbiota when compared to untreated or nitrate-only-treated microbiota.

## 1. Introduction

*Salmonella* is the second most frequent cause of foodborne illness in the United States, responsible for 896 outbreaks involving more than 23,000 illnesses between 2009 and 2015 [1]. From 2012 to 2019, there were 27 *Salmonella* outbreaks associated with contaminated beef, which affected 1103 individuals [2]. Zoonotic *Salmonella* infections occur mainly via oral exposure and subsequent ingestion of contaminated sources and, thus, in ruminants, the reticulorumen is the first major gastrointestinal habitat to be colonized by the ingested pathogen. Passage of *Salmonella* through pregastric compartments to the animal’s lower gastrointestinal tract can lead to the establishment of a carrier state upon subsequent translocation to the host interstitial and systemic environments [3]. Accordingly, from a food safety perspective, there is considerable interest in developing preharvest dietary interventions to prevent the ability of *Salmonella* to colonize and persist within animals. Feed additives delivered to the rumen are a practical approach to administer these interventions.

In the gastrointestinal tract, *Salmonella* spp. are believed to conserve energy for growth via fermentation or anaerobic respiration using oxidized compounds, such as nitrate, as terminal electron acceptors, and these acceptors may promote *Salmonella* colonization within the gut [4,5,6]. While anaerobic electron acceptors such as nitrate are generally limiting in the rumen, the consumption of high nitrate-containing heat- or drought-stressed forages, or the feeding of a methanogen-mitigating nitrate-based feed additive (SilvAir™, Cargill), to ruminants may favor *Salmonella* proliferation in the gut. In the lower gut, the production of reactive nitrogen compounds by the host can be induced by inflammation caused by the invading *Salmonella*; this can promote nitrate accumulation within the luminal and mucosal microenvironments as well as within tissues such as the spleen and liver [7,8]. Bacterial nitrate respiration in anaerobic gut environments primarily consumes electrons derived during catabolic processes for the enzymatic reduction of nitrate to nitrite and then to ammonia which drives the establishment of a proton gradient capable of supporting proton motive force phosphorylation [9,10]. In some cases, bacteria lacking F1·Fo-ATP synthases activity may indirectly conserve energy via the maintenance of an energetically favorable redox balance within the bacterial cell [9,10,11]. In many bacteria, including foodborne pathogens such as *Salmonella* and *Escherichia coli*, electrons flowing through the transport chain are consumed by a membrane-bound nitrate reductase (NarG) which catalyzes the reduction of nitrate to nitrite [9,10]. The nitrite is then further reduced to ammonia by the nitrite reductase isozymes NirB and NrfA [9,10]. In some cases, a periplasmic nitrate reductase (NapA) may also contribute to nitrate metabolism, and this has been reported to support *Salmonella* proliferation in the gut lumen [4,12]. Nitrate reductase activity is supported by molybdenum-associated enzymes such as those that interact with molybdenum-pyranopterin cofactors involved in reducing nitrate to nitrite [9,10,13]. Formate dehydrogenase, which is another molybdenum-associated enzyme, can also play a key role in contributing electrons derived from formate catabolism to the electron transport chain [9,10,13]. A constitutively expressed nitrate reductase, NarZ, may also contribute to nitrate reduction in *Salmonella* and *E. coli*, but its role is thought to facilitate the transition from aerobic to anaerobic respiration and is less dependent on molybdenum-containing enzymes [9,13].

As tungstate is an inhibitor of respiratory nitrate reductase activity, Zhu et al. [14] administered tungstate orally to mice and observed inhibition of a nitrate-respiring *Enterobacter cloacae* strain and wildtype *Escherichia coli* during in vitro culture in simulated gastrointestinal contents and in experimentally infected mice [14]. More recently, Yang et al. [15] reported significant inhibition of *E. coli* both during pure culture and co-culture with a *Bacillus coagulans* probiotic in a mucin-supplement broth supplemented with a tungstate product mixed with calcium. Mechanistically, in low molybdenum environments, tungstate is thought to be incorporated in place of molybdenum during synthesis of molybdenum-associated coenzymes, thereby resulting in the formation of nonfunctional nitrate reductase and formate dehydrogenase enzymes [9,10,13]. Accordingly, the objectives of this study were to (i) evaluate the effects of sodium tungstate on *Salmonella* growth when inoculated into mixed ruminal microbial cultures, (ii) determine the effect of tungstate on the growth of *Salmonella* serovars in pure culture, and (iii) assess its impact on nitrate and nitrite metabolism, methane production, and volatile fatty acid formation in ruminal microbial cultures.

## 2. Materials and Methods

### 2.1. Bacterial Strain

*Salmonella enterica* serovars Newport and Dublin used in this study were bovine isolates that had been acquired from fecal contents of cattle reared on a contemporarily managed New Mexico (USA) dairy [16]. *Salmonella* serovar Typhimurium (strain NVSL 95-1776) was originally isolated from a pig and was acquired from The United States Department of Agriculture’s National Veterinary Services (Ames, IA, USA). For routine culture and the pure culture experiments, the *Salmonella* strains were grown anaerobically in ½ strength BBL^TM^ Brain Heart Infusion (BHI) broth (Becton Dickinson and Company, Sparks, MD, USA). The ½ strength medium was used to avoid excessive acid production during fermentative growth and was prepared using ½ the amount of medium powder per volume recommended by the manufacturer. The prepared medium was distributed (9 mL/tube) to 13 × 100 mm glass culture tubes, sterilized in an autoclave, then aseptically equilibrated overnight to a 90% nitrogen: 5% carbon dioxide: 5% hydrogen atmosphere in a Bactron Anaerobic Chamber (Sheldon Manufacturing, Inc., Cornelius, OR, USA). After equilibration to the anaerobic gas phase, the culture tubes were closed aseptically with previously sterilized Hungate screw caps with fitted stoppers (Belco Glass, Vineland, NJ, USA).

### 2.2. Effect of Sodium Tungstate on Salmonella Growth in Cultures Containing Ruminal Microbiota

To evaluate effects of sodium tungstate treatment on *Salmonella* growth cocultured in the presence of ruminal microbiota, ruminal fluid was collected fresh the morning of the study from a healthy, 11-year old ruminally cannulated Jersey steer maintained on pasture consisting predominantly of Bermudagrass. Acquisition, surgery, care and use of the donor animal was approved by the Southern Plains Agricultural Research Center’s Institutional Animal Care and Use Experimental Animal Protocol #2016017 and complied with the Institutional Animal Care and Use Standard Operating Practices of the Southern Plains Agricultural Research Center, updated 7 April 2023. Rumen contents collected from the donor were withdrawn by hand from the opened cannula and immediately strained through a nylon paint strainer to remove large particles [17]. The strained fluid was collected in a 1500 mL container until full and then immediately closed to minimize exposure to atmospheric oxygen. The filtered fluid was transported to the lab within 30 min of collection. The pH of the ruminal fluid, measured upon arrival to the lab using a pH meter, was 6.18. Ruminal fluid (500 mL) was then mixed with 100 mL of anaerobic dilution solution [18] supplemented with 30 mM sodium formate while flushed with a constant flow of carbon dioxide. The resultant suspension was then inoculated with 10 µL of *S.* Newport that was cultivated overnight in nitrate-lacking ½ BHI broth and then randomly distributed (9 mL/tube) to 18 × 150 mm crimp top tubes preloaded with 0.2 g ground alfalfa. To facilitate quantitative recovery of the *S.* Newport used as a challenge organism in this mixed culture study, the *S.* Newport strain, naturally resistant to novobiocin, was previously made resistant to 20 µg nalidixic acid/mL via serial culture in medium supplemented with gradually increasing amounts of the antibiotics as described with another *Salmonella* [19]. While the Clinical Laboratory and Standards Institute (CLSI) does not publish resistance breakpoints for *Salmonella*, the minimum inhibitory concentration (MIC) of nalidixic acid against the resistant *S.* Newport, as determined using Sensititre Gram Negative NARMS Plates (TREK Diagnostics Inc., Oakwood Village, OH, USA) was equal to or exceeded 32 µg/mL. This value is the National Antimicrobial Resistance Monitoring System breakpoint for bacteria considered to be resistant to this antibiotic. Incubation tubes were closed upon addition of the rumen fluid suspensions, and treatments of sodium tungstate and sodium nitrate were administered by injecting small volumes (<1.0 mL) of separate stock solutions of tungstate, nitrate (1000 mM each) or water to achieve 100 mM tungstate and 5 mM nitrate. The tungstate dose was chosen based on the highest dose used by Gates et al. [20] which reported good inhibitory effect against *E. coli*. The nitrate dose was selected to promote induction of respiratory nitrate metabolism and provide a sufficient amount to serve as an alternative electron acceptor able to consume electrons at the expense of methanogenesis [21]. Stock solutions were prepared in sterile 13 × 100 mm Hungate tubes loaded with filter-sterilized ½ BHI broth (0.2 µm Whatman Puradisc^TM^ 25 mm filter, Global Life Sciences Solutions Operations UK Ltd., Buckinghamshire, UK). The tubes containing the sterilized stock solutions were closed with presterilized screw caps fitted with rubber stoppers and then flushed aseptically for 20 min with 100% carbon dioxide passed through separate freshly opened 0.2 µm Whatman Puradisc^TM^ 25 mm filters before use. Injections were administered via 1 mL syringes affixed to 21-gauge needles. All tubes were then incubated for 26 h at 39 °C in an Innova 4000 Digital Incubator Shaker (New Brunswick Scientific, Edison, NJ, USA) while agitated at 43 rpm.

### 2.3. Effect of Sodium Tungstate Supplementation on the Growth of Salmonella Under Pure Culture Conditions

For pure culture tests, treatments were administered to the ½ BHI broth prior to inoculation via addition of small volumes (<1.0 mL) of water or concentrated stock solutions of 150 mM nitrate, nitrite, tungstate or tungsten (prepared with sodium salts as described earlier). Tungsten, in its reduced state, was used as a non-oxygen containing alternative to tungstate. These additions were calculated to achieve upon initiation of incubation 5 mM nitrate and a lesser dose of 50 mM tungstate, or tungsten, than that used with the mixed rumen microbiota. Test cultures were inoculated with 0.2 mL of each respective strain obtained from cultures grown overnight in ½ BHI broth supplemented without or, in the case of the nitrate-adapted *S.* Newport population, with 5 mM sodium nitrate; the latter was used to provide an inoculum having been grown in the presence of nitrate immediately prior to its use as inoculum for the present study. All cultures were incubated at 39 °C as described earlier.

### 2.4. Analytical Methods

For the mixed culture study with freshly collected rumen microbes, the experimentally inoculated *S.* Newport as well as wildtype coliforms and lactic acid bacteria within the rumen microbiota populations were enumerated via serial dilution and plating of collected fluid to Difco Brilliant Green (Becton Dickinson and Company) agar supplemented with 25 µg novobiocin and 20 µg nalidixic acid/mL and *E. coli*/coliform or Lactic Acid Bacteria Petrifilm (3MTM Petrifilm^TM^, St. Paul, MN, USA), respectively. For the pure culture studies, growth was measured as changes in optical density (OD) at 600 nm using a spectrophotometer (Spectronic 20D, Thermo Spectronic Inc., Madison, WI, USA). Mean specific growth rates were calculated during logarithmic growth using the equation (ln OD time 2 − ln OD time 1)/(time 2 − time 1).

For the mixed culture study, gas production was measured at the end of the incubation period by volume displacement, and gas composition was measured by gas chromatography [22]. For the mixed rumen population incubations and the pure culture experiments, fluid samples (0.5 mL) collected upon initiation of incubation as well as at intervals as indicated during incubation were frozen until subsequent colorimetric analysis for measurement of accumulations of ammonia, nitrate and nitrite [23,24,25]. Moreover, six different volatile fatty acids including acetate, propionate, butyrate, isobutyrate, valerate and isovalerate were measured in fluid samples (approximately 3 mL) collected at the beginning and end of the 26 h in vitro mixed culture incubation. Briefly, the samples were mixed using a vortex (VWR^®^ digital vortex mixer, Radnor, PA, USA) and centrifuged (Clinical 100 Laboratory Centrifuge, VWR) at 2000× *g* for 20 min and then filtered through a 0.45 µm pore size filter. The resultant supernatant fluids were analyzed using an Agilent 6890 N gas chromatograph (Agilent Technologies Inc., Wilmington, DE, USA) equipped with an FID, a fused silica column (Supelco^®^, Nukol 15 m × 0.53 m × 0.5 µm, Millipore, Burlington, MA, USA) and a 7683 series auto-injector according to a previously used method [26,27]. Volatile fatty acid concentrations are presented as the difference between 26 h and initial concentrations and thus reflect net accumulations. Stoichiometric estimates of amounts of hexoses fermented during the 26 h incubations were calculated from molar values of ½ acetate + ½ propionate + butyrate + valerate [28].

### 2.5. Statistical Analysis

In the mixed and pure culture experiments, all treatments were conducted in triplicate and because the respective microbial populations within each tube had the opportunity to respond independently from populations in other tubes, each incubation tube was considered an individual experimental unit. An *n* of 3 for each treatment was chosen based on previous in vitro studies with gut microbiota indicating sufficient statistical power to detect relevant differences [19,21]. Growth curves and concentrations of nitrate and ammonia measured at each sampling time are presented graphically.

In the mixed culture experiments, log_10_ transformations of bacterial counts, final pH and net accumulations of methane, hydrogen, volatile fatty acids and ammonia after 26 h were analyzed for effects of treatments (none, nitrate, tungstate or nitrate plus tungstate) using a general analysis of variance. Amounts of nitrate consumed and nitrite accumulated after 6 and 26 h were analyzed similarly. When significant, means were compared using an LSD multiple comparison test. All analyses were conducted using STATISTIX Version 10 Analytical Software (Tallahassee, FL, USA).

For the pure culture experiment testing nitrate-naive and nitrate-adapted *S.* Newport populations, maximum OD, mean specific growth rates and ammonia concentrations were analyzed for main effects of treatment (none, nitrate, tungstate or nitrate plus tungstate), nitrate adaptation (nitrate-naive or nitrate-adapted) or their interaction using a general analysis of variance. Initial rates of nitrate metabolism (calculated as the difference between concentrations measured at 0 and 4 h incubation) and peak nitrite concentrations were analyzed similarly but only for treatments that included additions of nitrate. Additionally, because nitrite is a transitory intermediate produced during anaerobic metabolism of nitrate, the total amount of nitrite consumed (calculated as the difference between the amount of nitrate catabolized minus the residual amount of nitrite measured at the end of the 12 h incubations) was also analyzed.

Non-nitrate adapted *S.* Dublin, *S.* Typhimurium and *S.* Newport cultured with nitrate, tungstate (or tungsten) or their combination were analyzed as described above except omitting adaptation as an independent variable and its interaction with tungstate or tungsten.

## 3. Results and Discussion

### 3.1. Sodium Tungstate Promoted Salmonella Growth and Altered Fermentation Profiles During Culture with Mixed Ruminal Microbiota

**Figure 1:**
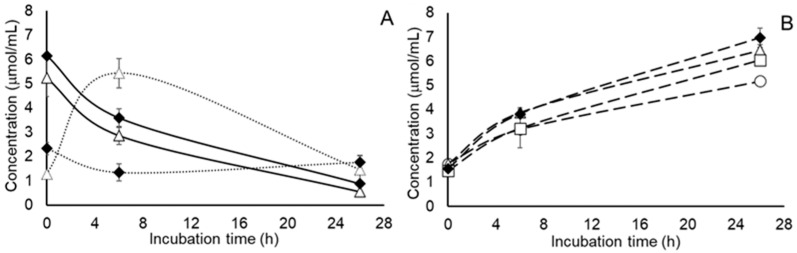
Consumption of nitrate (solid lines) and accumulation of nitrite (dotted lines) (Graph (**A**)) or accumulations of ammonia (Graph (**B**)) during culture of non-adapted *Salmonella enterica* serovar Newport with mixed populations of freshly collected rumen fluid without nitrate or tungstate treatment (circles), or with 100 mM added tungstate alone (open squares), 5 mM nitrate alone (triangles) or their combination (filled diamonds). Values are least squares means ± standard deviations from *n* = 3 cultures per treatment. Statistical comparisons of rates of nitrate metabolism, peak nitrite concentrations are presented in Table 1 or the text.

Results from our mixed culture study with freshly collected populations of rumen microbes revealed effects of treatment on the recovery of experimentally inoculated *S.* Newport as well as wildtype populations of total coliforms and lactic acid bacteria after 26 h of incubation but not after 6 h of incubation (Table 1). In all cases, the bacterial populations measured in the present study were higher after 26 h incubation in cultures treated with tungstate, whether alone or combined with nitrate, than in untreated cultures or cultures treated with nitrate alone. These observations conflict with the approximately 2-log_10_ fold reductions in viable *E. cloacae* and *E. coli* recovered from gastrointestinal contents of tungstate-treated mice [14]. Residual nitrate concentrations after 26 h incubation were slightly higher (*p* = 0.0041; SEM = 0.039) in the nitrate-supplemented populations treated with tungstate than those not treated with tungstate (Figure 1). Rates of nitrate metabolism (*p* = 0.1603, SEM = 0.003) and amounts of nitrate catabolized (*p* = 0.2588; SEM = 0.313) did not differ in the present study (Table 1); averaging 0.175 µmol/mL per h and 4.98 µmol/mL, respectively. However, as illustrated in Figure 1, a marked reduction in nitrite accumulation (*p* = 0.0003, SEM = 0.310) was observed in the tungstate-treated populations after 6 h of incubation but not after 26 h of incubation (*p* = 0.3202, SEM = 0.163). Our findings are consistent with those of Marais et al. [29] who reported a modest inhibitory effect of tungstate on nitrate degradation by mixed populations of rumen microbes but an appreciable reduction in nitrite accumulation. Considering that the *nirB* nitrite reductase is maximally expressed at nitrate concentrations exceeding 2 µmol/mL and the expression of periplasmic *nrfA* nitrite reductase activity increases from 70% of its maximal expression to near 100% at 1 µmol nitrate/mL or less, it is reasonable to suspect that both isozymes are contributing to nitrite metabolism, albeit at different times [30]. Nitrite concentrations measured at the start of the incubation (Figure 1) differed by approximately 1 µmol/mL (*p* = 0.0024; SEM = 0.107) but it is not apparent that this difference, which may be due to differences in nitrite uptake by the different populations, would have had a major impact on the expression of the different nitrite reductase isozymes. It is known, for instance, that nitrate rather than nitrite is the major regulator of nitrite reductase activity [30]. Ammonia accumulations (Figure 1) after 26 h incubation of the mixed populations were, as expected, higher (*p* = 0.0001; SEM = 0.139) for populations supplemented with nitrate than populations not supplemented with nitrate as the ultimate fate of nitrate and subsequent nitrite metabolism within mixed populations of rumen microbes is ammonia [31].

**Figure 2:**
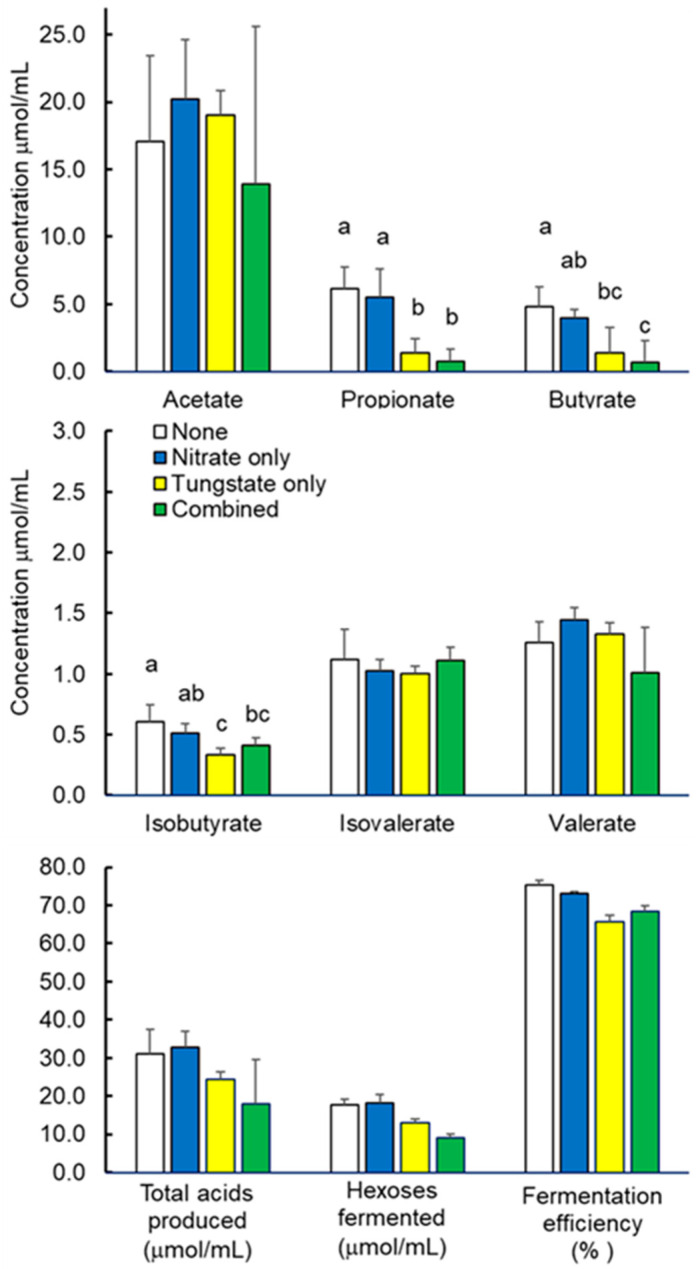
Fermentation characteristics of mixed populations of freshly collected rumen fluid treated without (white bars) or with 5 mM nitrate alone (blue bars), 100 mM tungstate alone (yellow bars) or their combination (green bars). Bars are least squares means ± standard deviations from *n* = 3 cultures per treatment and those associated with unlike lower case letters differ (*p* < 0.05). Statistical comparisons of respective volatile fatty acid accumulations, presented as net amounts produced, and stoichiometric estimates of amounts of hexoses fermented and fermentation efficiency are discussed further in the text.

Analysis of fermentation characteristics during incubation of the ruminal microbiota are presented in Table 2 and Figure 2. These results reveal treatment effects on methane accumulations but not on hydrogen accumulations. When compared to untreated controls, methane accumulations decreased by 51 to 75%, with the decrease being greater in incubations treated with the combination of nitrate and tungstate than by nitrate or tungstate alone (Table 2). A treatment effect was also observed on net accumulations of the fermentation acids propionate (*p* = 0.0042, SEM = 0.865), butyrate (*p* = 0.0270, SEM = 0.864) and isobutyrate (*p* = 0.0257, SEM = 0.052) but not on acetate (*p* = 0.7222, SEM = 4.092), valerate (*p* = 0.1716, SEM = 0.126) or isovalerate (*p* = 0.6972. SEM = 0.084) (Figure 2). With respect to the untreated and nitrate-only-treated populations, accumulations of methane and volatile fatty acids are reflective of accumulations observed in other studies with mixed populations of ruminal microbes [21,29]. However, the dramatic decreases in propionate, butyrate and isobutyrate in the populations treated with tungstate, whether with or without added nitrate, suggests an appreciable inhibition of carbohydrate fermentation in the ruminal cultures treated with tungstate alone or when combined with nitrate. This finding is supported by an observed tendency (*p* = 0.038, SEM = 2.423) for a decrease in stoichiometric estimates of amounts of hexose fermented in the mixed cultures treated with tungstate, which were more than 27 and 49% lower than those for cultures not treated with tungstate (Figure 2). Overall, the markedly lower accumulations of methane, propionate, butyrate and isobutyrate in the populations incubated with added tungstate, whether alone or combined with nitrate, compared to those populations not treated with tungstate suggests, but does not prove, a potential oxygenation or perturbation of redox homoeostasis within the tungstate treated populations. Conceptually, oxygen derived from tungstate could be utilized as a terminal electron acceptor by oxygen-respiring bacteria such as *Salmonella* and *E. coli* as well as by facultatively aerobic lactic acid bacteria potentially present within the rumen microbiota. Consequently, electrons generated during fermentative metabolism would be expected to be redirected away from the reduction of carbon dioxide to methane to the more thermo-dynamically favorable reduction of oxygen to water similar to the process of electrons being redirected away from methane production to the respiratory reduction of nitrate to ammonia. Likewise, the decreased production of propionate, butyrate and isobutyrate was also expected as each of these fatty acids often serve as electron sinks within fermentative environments. Tungstate-derived oxygen could potentially increase the redox potential within the otherwise anaerobic rumen incubations which, by itself, could be detrimental to the fermentative production of methane and volatile fatty acids. However, an increase in redox potential by itself would not necessarily be expected to appreciably affect volatile fatty acid accumulations as Marais et al. [29] reported that high redox potentials resulting from high accumulations of nitrite had modest effects on digestion and volatile fatty acid accumulation. Their findings are supported by the results of our study showing little if any difference in volatile fatty acid accumulations between non-treated and nitrate-only-treated populations of ruminal microbes, the latter achieving accumulations exceeding 5 mM nitrite. In the present study, fermentation efficiency (Figure 2), which reflects the caloric energy recovered in the major fermentation acids acetate, propionate and butyrate relative to the caloric energy available in glucose, was unaffected by treatment (*p* = 0.1467, SEM = 2.853).

### 3.2. Sodium Tungstate Promoted Growth of Salmonella Serovars During Pure Culture

**Figure 3:**
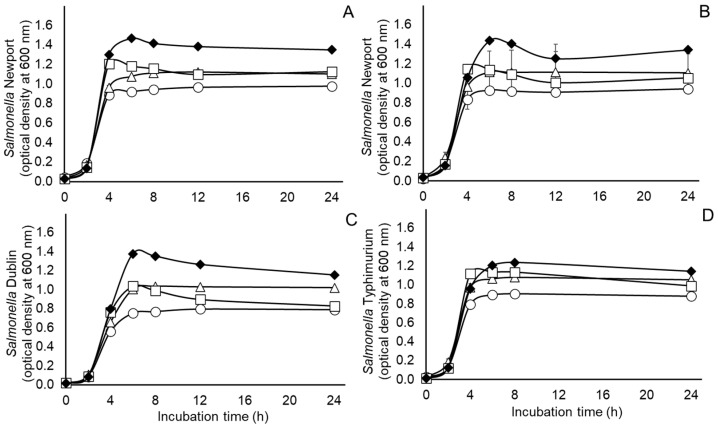
Growth of non-adapted (**A**) or nitrate-adapted (**B**) *Salmonella enterica* serovar Newport and non-adapted serovars Dublin (**C**) and Typhimurium (**D**) during pure culture in ½ strength Brain Heart Infusion broth supplemented without (circles) or with 50 mM added tungstate (squares), 6 mM added nitrate (triangles) or their combination (filled diamonds). Values are least squares means ± standard deviations from *n* = 3 cultures per treatment. Statistical comparisons of maximum optical densities and mean specific growth rates are presented in Table 3 or Table 4.

In light of the unexpected growth-promoting effect of tungstate observed on the bacterial populations measured in the experiment with the populations of ruminal microbes, the following studies were conducted to observe if the effect may occur similarly during pure culture of three representative *Salmonella* serovars, Newport, Dublin and Typhimurium. Growth curves of nitrate-adapted and nitrate-naïve *S.* Newport grown in ½ strength BHI broth treated without or with 5 mM nitrate, 50 mM tungstate or their combination are presented in Figure 3A,B. As presented in Table 3, the main effects of treatment, adaptation and their interaction were observed on maximum OD achieved by the nitrate-adapted and non-nitrate-adapted *S.* Newport cultures (Table 3). In this case, ODs were highest in cultures treated with the combination of nitrate and tungstate, intermediate in the cultures treated with nitrate or tungstate alone and lowest in the untreated control cultures. Similarly, growth curves of *S.* Dublin and Typhimurium are presented in Figure 3C,D. Significant treatment effects on maximum OD were observed for the *S.* Dublin and Typhimurium cultures, with OD being highest for cultures treated with the combination of nitrate and tungstate, intermediate in cultures treated with nitrate or tungstate alone and lowest for untreated control cultures (Table 4).

With respect to mean specific growth rates, a treatment by adaptation interaction, but not the main effects of treatment or of adaptation, was observed on mean specific growth rates of the nitrate-adapted and non-nitrate-adapted *S.* Newport (Table 3). Mean specific growth rates were highest for the non-nitrate-adapted *S.* Newport culture treated with the combination of nitrate and tungstate and lowest for the untreated non-nitrate-adapted *S.* Newport although these growth rates did not necessarily differ significantly between the different cultures (Table 3). Likewise, mean specific growth rates for populations of *S.* Dublin as well as *S.* Typhimurium were higher for cultures treated with the combined treatment of nitrate plus tungstate or treated with tungstate alone than untreated cultures or cultures treated with nitrate alone, but again the differences were not always significant (Table 4). These findings indicate that, when compared to the untreated control cultures, available energy substrates in the medium were not limiting for any of the populations but rather were in excess in the treated cultures. For instance, an increase in the amount of growth, as reflected by increased maximum OD, was expected during anaerobic respiration with nitrate. Conversely, the dramatic increase in growth observed in cultures grown with added tungstate, whether alone or with added nitrate, was unexpected as tungstate is reported to be inhibitory to respiratory nitrate metabolism [14,20]. Accordingly, results from the present pure culture studies conflict with earlier reports of appreciable growth inhibition of nitrate-respiring *Paracoccus*, *Escherichia*, *Proteus* and *Enterobacter* strains cultured with 0.10 to 10 mM tungstate [14] and of an NAP-expressing *E. coli* treated with tungstate at levels exceeding 15 mM [20]. The contrasting results between the present and earlier research may be due to differences in cultural conditions used in the respective studies. For instance, Gates and colleagues [20] grew their *E. coli* in a minimal medium containing salts, about 0.006% glycerol, 0.05% casein hydrolysate, 0.002% thiamine, 1 uM selenite and sodium tungstate, the latter replacing ammonium heptamolybdate. Zhu et al. [14] conducted their anaerobic growth assays with tungstate using a no-carbon E medium supplemented with 0.5% pig-sourced mucin, trace elements (containing molybdenum) and additions (40 mM each) of sodium formate, sodium nitrate, dimethyl sulfoxide and trimethylamine N-oxide, substrates that are also catabolized by molybdenum dependent enzymes [13]. In the pure cultures of the present study, formate was not supplemented to the ½ BHI broth and no attempts were made to control the molybdenum content of the anaerobic ½ BHI broth. Moreover, reducing agents such as cysteine-HCl or dithiothreitol were not added to the ½ BHI broth used here and thus it is possible that oxygen or oxygen radicals potentially released into the medium would be available as electron acceptors for respiration. Accordingly, it seems reasonable to hypothesize that the enhanced growth observed presently may be due to oxygenation of the medium resulting from the addition of 50 mM tungstate to the medium in this study which could thus provide additional respiratory electron acceptors energetically more favorable than nitrate. This hypothesis, being outside the scope of the present study, has yet to be tested.

**Figure 4:**
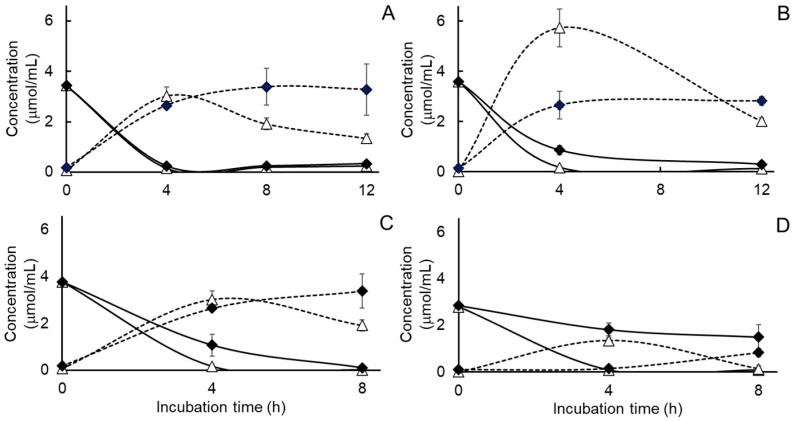
Consumption of nitrate (solid lines) and accumulation of nitrite (short-dashed lines) during pure culture of non-adapted (**A**) or nitrate-adapted (**B**) *Salmonella enterica* serovar Newport and non-adapted serotypes Dublin (**C**) and Typhimurium (**D**) in ½ strength Brain Heart Infusion broth supplemented with 5 mM added nitrate and without (triangles) or with (filled diamonds) 50 mM added tungstate. Values are least squares means ± standard deviations from *n* = 3 cultures per treatment. Statistical comparisons of rates of nitrate metabolism and peak nitrite concentrations are presented in Table 3 and Table 4.

**Figure 5:**
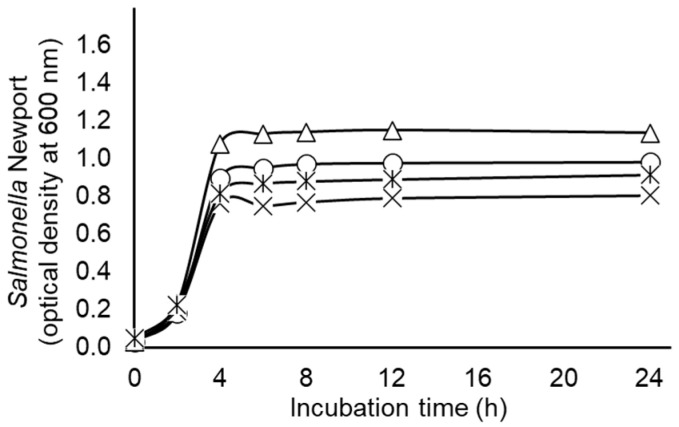
Growth of non-nitrate-adapted *Salmonella enterica* serovar Newport during pure culture in ½ strength Brain Heart Infusion broth supplemented without (circles) or with 50 mM added tungsten (X), 6 mM added nitrate (triangles) or their combination (bar X). Values are least squares means ± standard deviations from *n* = 3 cultures per treatment. Statistical comparisons of maximum optical densities and mean specific growth rates are presented textually within the results section.

With respect to the effects of tungstate on nitrate metabolism, the main effects of treatment, adaptation and their interaction were observed on the *S.* Newport populations, but the rate of nitrate catabolism was only decreased in cultures that had previously been adapted to nitrate (Table 3). The differences in rates of nitrate metabolism were modest however (Figure 4A,B) and possibly reflect a more rapid onset of tungstate-sensitive, molybdenum-associated enzyme synthesis by the nitrate-adapted than by the non-adapted *S.* Newport populations (Table 3). As illustrated in Figure 5, a main effect of treatment but not of adaptation nor a treatment by adaptation interaction were observed on total amounts of nitrate metabolized after 12 h incubation by the nitrate-adapted and non-adapted *S*. Newport cultures, (Table 3). Means for the main effect of tungstate on amounts of nitrate metabolized after 12 h were 3.33 versus 3.20 µmol nitrate/mL for cultures treated without or with tungstate, respectively. Peak accumulations of nitrite were higher in the nitrate-adapted cultures incubated with additions of nitrate alone than the other cultures (Table 3), and as illustrated in Figure 4A,B, peak nitrite concentrations declined appreciably only in the *S.* Newport cultures not supplemented with added tungstate. The main effects of treatment, but not of prior nitrate adaptation or the respective interaction, were observed on the calculated amounts of nitrite metabolized (Table 3). In this case, appreciably more nitrite was metabolized in the *S.* Newport cultures not treated with tungstate than those treated with tungstate, with means for the main effect of tungstate treatment being 3.96 and 2.17 µmol nitrite/mL, respectively.

For the *S.* Dublin and Typhimurium cultures, the disappearance of nitrate and accumulation of nitrite during the 8 h incubation are illustrated in Figure 4C,D. Significant or near-significant treatment effects were observed on initial rates and amounts of nitrate metabolized by *S.* serovars Dublin and Typhimurium, with nitrate being metabolized less rapidly and less nitrate being metabolized by the cultures treated with the combination of nitrate plus tungstate than the cultures treated with nitrate alone (Table 4). Peak accumulations of nitrite were higher in the *S.* Typhimurium cultures treated with nitrate alone than those treated with the combination of nitrate and tungstate but were unaffected by treatment in the *S.* Dublin cultures (Table 4). Nevertheless, when calculated as the difference between the amount of nitrate metabolized and amount of residual nitrite measured after the 8 h incubation, more nitrite was metabolized in the *S.* Dublin and Typhimurium cultures treated with nitrate alone than those treated with the combination of nitrate and tungstate (Table 4).

A treatment effect was observed on net ammonia accumulations for all *Salmonella* cultures, with net accumulations of ammonia being higher in the cultures that contained added nitrate, whether alone or in combination with tungstate, than those not containing nitrate (Table 3 and Table 4). Considering that nitrite and ammonia accumulations represent amounts that are produced and consumed during microbial metabolism it is possible that the *Salmonella* populations cultured with additions of nitrate, whether alone or with tungstate, may have consumed at least some of the nitrite and ammonia derived from nitrite for other catabolic or anabolic purposes.

To test the effects of tungsten, which in its reduced state exists as an element devoid of oxygen, on the growth of anaerobically grown *S.* Newport, the non-nitrate adapted strain was cultured in anaerobic ½ BHI supplemented without or with additions of 5 mM nitrate, 50 mM tungstate, 50 mM tungsten or the combination of nitrate and tungsten. Analysis of growth curves presented in Figure 5 revealed significant treatment effects on maximum OD (*p* < 0.0001; SEM = 0.087) and mean specific growth rates (*p* < 0.0001; SEM = 0.019). For instance, maximum OD were lowest for *S.* Newport cultured with tungsten alone or in combination with nitrate (0.789 and 0.891, respectively), highest for cultures treated solely with nitrate or tungstate (1.150 and 1.127, respectively) and intermediate for untreated controls (0.979). Similarly, mean specific growth rates were slowest for cultures treated with tungsten alone or when combined with nitrate (0.694 and 0.646 per h, respectively), most rapid for cultures treated with tungstate alone (0.888 per h) and intermediate for untreated cultures or nitrate-only-treated cultures (0.800 and 0.774 per h, respectively). These results support the hypothesis that oxygen associated with tungstate may be contributing to the promotion of bacterial growth observed in the tungstate-treated cultures.

**Figure 6:**
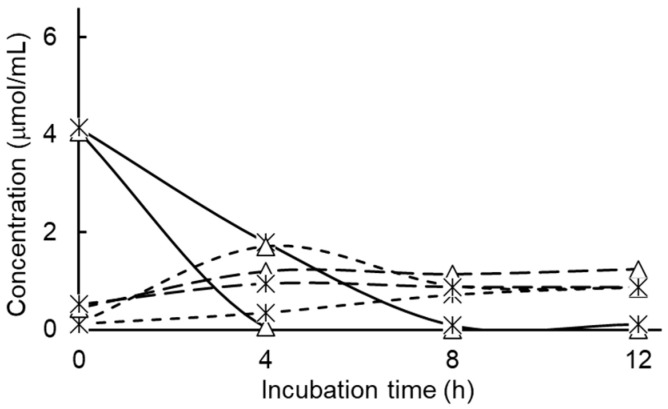
Consumption of nitrate (solid lines) and accumulations of nitrite (short-dashed lines) or ammonia (long-dashed lines) during pure culture of non-adapted *Salmonella enterica* serovar Newport in ½ strength Brain Heart Infusion broth treated without or with 50 mM added tungsten, 6 mM added nitrate (triangles) or their combination (bar X). Values are least squares means ± standard deviations from *n* = 3 cultures per treatment. Statistical comparisons of rates of nitrate metabolism and peak nitrite concentrations are presented textually within the results section.

Results for the effects of tungsten on nitrate metabolism are presented in Figure 6. Significant effects were observed with tungsten treatment, whether alone or combined with nitrate, on rates on nitrate metabolism (*p* = 0.0001, SEM = 0.058) and amounts of nitrate metabolized (*p* = 0.0001, SEM = 0.117). Initial rates of nitrate metabolism measured during the first 4 h of incubation (2.95 versus 1.69 µmol/mL per h, respectively) and amounts of nitrate metabolized (1.72 versus 0.36 µmol/mL, respectively) were more rapid for cultures treated with nitrate alone than those treated with nitrate combined with tungsten. Peak nitrite accumulations were similarly affected by treatment (*p* = 0.0227, SEM = 0.267), with accumulations being higher for *S.* Newport cultures treated with nitrate alone than those treated with nitrate combined with tungsten (1.72 versus 0.36 µmol/mL, respectively), although a treatment effect on amounts of nitrite metabolized (*p* = 0.6389, SEM = 0.105) was not observed, averaging 4.10 µmol nitrite metabolized per mL. These results clearly support the concept that tungsten is inhibitory to nitrate reductase and provide evidence that the growth-promoting effect of tungstate, at the concentrations used here, may be due to the oxidized state of the element.

While the present work has implications pertaining to *Salmonella* colonization within the rumen, which is a reservoir of importance for dietary preharvest food safety interventions, additional research is needed to fully elucidate effects of tungstate on core and functional microbiota in the rumen as well as other important colonization sites. This would include in vitro and in vivo research with microbiota residing within the lower gut as well as within the host lymphatic tissues of ruminants, mice and other relevant monogastric species as these sites are recognized as major gastrointestinal and systemic sites of colonization, each harboring their own distinct microbial populations. Moreover, additional research is needed to more clearly elucidate kinetic and thermodynamic drivers of nitrate and nitrite metabolism and potential effects on the microbial diversity within the respective environments as the possible mechanisms discussed within this report are speculative at best. Another limitation of the present study is that the tungstate dose used, while based on amounts used by Gates et al. [20], appears to have been higher than needed and would likely exceed those expected to be encountered in practical animal feeding or treatment situations.

## 4. Conclusions

Contrary to earlier reports suggesting that tungstate treatment may be a promising approach to reduce gut colonization by nitrate-respiring Enterobacteriaceae, results from the present study indicated that 50 mM sodium tungstate treatment, whether alone or in combination with 5 mM nitrate, increased the amount of growth of anaerobically grown *Salmonella* serovars Newport, Dublin and Typhimurium. Results from this study further indicate that increased growth of experimentally inoculated *S.* Newport as well as wildtype populations of *E. coli* and lactic acid bacteria occurred during in vitro incubation with mixed populations of freshly collected ruminal microbes treated with 100 mM tungstate when compared to non-tungstate-treated incubations. Effects of tungstate treatment on nitrate and nitrite metabolism were as expected, however, with nitrate and nitrite metabolism being decreased during pure culture. Similarly, nitrite metabolism, but not nitrate metabolism, by mixed populations of freshly collected rumen microbes was decreased by tungstate treatment when compared to non-tungstate-treated incubations, but this is also consistent with earlier reports with rumen populations. When cultured with reduced tungsten rather than tungstate (each at 50 mM), an inhibitory effect on *S.* Newport was observed and effects on nitrate and nitrite metabolism were as expected, thus suggesting that in the present study tungstate treatment may have served as a source of reducible oxygen able to complement respiratory energy conservation. Clearly, additional research is warranted to better understand the conditions needed to optimize the antimicrobial effect of tungstate, or perhaps preferably tungsten, treatment against nitrate-respiring pathogens.

## Figures and Tables

**Table 1:** Effect of nitrate, tungstate or their combination on select microbial populations after in vitro incubation of freshly collected rumen microbiota.

Populations ^3^	Treatment ^1^					
	Untreated	Nitrate	Tungstate	Combined	*p* Value ^2^	SEM ^2^
*Salmonella* Newport (log_10_ CFU/mL)						
After 6 h	3.46	3.82	3.49	3.77	0.0516	0.093
After 26 h	2.84 ^b^	3.30 ^b^	6.54 ^a^	6.43 ^a^	0.0003	0.426
Total coliforms (log_10_ CFU/mL)						
After 6 h	4.30	4.28	3.95	4.33	0.1139	0.106
After 26 h	3.08 ^c^	4.01 ^b^	6.90 ^a^	6.79 ^a^	<0.0001	0.180
Lactic acid bacteria (log_10_ CFU/mL)						
After 26 h	6.87 ^b^	7.13 ^b^	7.97 ^a^	7.86 ^a^	0.0002	0.108

^1^ Nitrate concentrations, when administered alone or combined with tungstate, at the beginning of the incubation period were approximately 5.69 ± 0.70 µmol/mL mM. Tungstate was added to achieve 100 mM when administered alone or combined with nitrate. ^2^ Results from a general analysis of variance with an LSD separation of means. SEM, standard error of the mean. ^3^ Populations of *S.* Newport, total coliforms and lactic acid bacteria at time 0 were 4.01 ± 0.07, 4.64 ± 0.04 and 7.34 ± 0.09 log_10_ CFU/mL, respectively. ^a,b,c^ Least squares means within rows with unlike superscript differ (*p* < 0.05).

**Table 2:** Effect of nitrate, tungstate or their combination on fermentation gas, pH and nitrate and nitrite characteristics during in vitro incubation of freshly collected rumen microbiota.

	Treatment ^1^					
Measurements	Untreated	Nitrate	Tungstate	Combined	*p* Value ^2^	SEM ^2^
Hydrogen (µmol/mL)	0.04	0.02	0.01	0.02	0.2411	0.011
Methane (µmol/mL)	26.90 ^a^	13.02 ^b^	12.64 ^b^	6.60 ^b^	0.0008	2.073
pH	5.73 ^b^	5.87 ^b^	7.32 ^a^	7.40 ^a^	<0.0001	0.055
Rate of nitrate metabolism after 26 h (µmol/mL per h)	NA ^3^	0.173	NA	0.166	0.1603	0.003
Nitrate consumed after 6 h (µmol/mL)	NA	2.19	NA	2.08	0.8155	0.313
Nitrate consumed after 26 h (µmol/mL)	NA	4.47 ^a^	NA	4.29 ^b^	0.0259	0.038
Nitrite accumulation after 6 h (µmol/mL)	NA	4.15 ^a^	NA	0.01 ^b^	0.0003	0.310
Nitrite accumulation after 26 h (µmol/mL)	NA	0.33	NA	0.07	0.3202	0.163

^1^ Nitrate concentrations, when administered alone or combined with tungstate, at the start of incubation were 5.69 ± 0.70 µmol/mL. Tungstate was added to achieve 100 mM when administered alone or combined with nitrate. ^2^ Results are from a general analysis of variance with an LSD separation of means. SEM, standard error of the mean. ^3^ NA, not applicable. ^a,b^ Least square means within rows with unlike superscript differ (*p* < 0.05).

**Table 3:** Effect of nitrate, tungstate or their combination on cultural characteristics and nitrogen metabolism during pure culture of *Salmonella* Newport populations without (non-adapted) or with (nitrate-adapted) immediate prior nitrate exposure.

	Treatments ^1^				*p* Values ^2^			
	None	Nitrate	Tungstate	Combined	Treatment	Adaptation	Interaction	SEM ^2^
Maximum optical density (600 nm)								
Non-adapted *S.* Newport	0.98 ^f^	1.12 ^e^	1.21 ^c^	1.47 ^a^	<0.0001	<0.0001	0.0015	0.006
Nitrate-adapted *S.* Newport	0.94 ^g^	1.11 ^e^	1.14 ^d^	1.44 ^b^				
Mean specific growth rate (per h)								
Non-adapted *S.* Newport	0.78 ^c^	0.87 ^bc^	0.93 ^ab^	0.97 ^a^	0.1070	0.7992	0.0294	0.034
Nitrate-adapted *S.* Newport	0.89 ^ab^	0.89 ^ab^	0.89 ^ab^	0.86 ^bc^				
Rate of nitrate consumption (µmol nitrate/mL per h)								
Non-adapted *S.* Newport	---	1.64 ^ab^	---	1.59 ^b^	0.0001	0.0222	0.0005	0.027
Nitrate-adapted *S.* Newport	---	1.71 ^a^	---	1.37 ^c^				
Total nitrate consumed by 12 h (µmol nitrite/mL)								
Non-adapted *S.* Newport	---	3.19	---	3.10	0.0101	0.0003	0.3116	0.040
Nitrate-adapted *S.* Newport	---	3.48	---	3.30				
Peak nitrite produced (µmol nitrite/mL)								
Non-adapted *S.* Newport	---	3.03 ^b^	---	3.38 ^b^	0.0041	0.0102	0.0010	0.321
Nitrate-adapted *S.* Newport	---	5.73 ^a^	---	2.82 ^b^				
Total nitrite consumed by 12 h (µmol nitrite/mL) ^3^								
Non-adapted *S.* Newport	---	4.14	---	1.93	0.0010	0.8677	0.2570	0.353
Nitrate-adapted *S*. Newport	---	3.78	---	2.42				
Rate of ammonia accumulation (µmol/mL per h)								
Non-adapted *S.* Newport	0.02	0.06	0.02	0.08	0.0002	0.5808	0.4557	0.012
Nitrate-adapted *S.* Newport	0.01	0.06	0.01	0.09				
Net ammonia accumulation (µmol/mL)								
Non-adapted *S.* Newport	0.23	0.80	0.18	0.80	0.0001	0.3879	0.1985	0.198
Nitrate-adapted *S.* Newport	0.21	0.73	0.14	1.20				

^1^ Nitrate concentrations, when administered alone or combined with tungstate, at the start of incubation were 3.44 ± 0.36 and 3.59 ± 0.88 µmol/mL for non-adapted and nitrate-adapted cultures, respectively. Tungstate was added to achieve 50 mM when administered alone or combined with nitrate. ^2^ Results are from a general analysis of variance with an LSD separation of means. SEM, standard error of the mean. ^3^ Calculated as the difference between amount of nitrate catabolized and residual nitrite measured after 12 h incubation. ^a–g^ Least square means within rows with unlike superscript differ (*p* < 0.05).

**Table 4:** Effect of nitrate, tungstate or their combination on cultural characteristics and nitrogen metabolism during pure culture of Salmonella serovars Dublin and Typhimurium.

	Treatments ^1^					
	None	Nitrate	Tungstate	Combined	*p* Values ^2^	SEM ^2^
Maximum optical density at 600 nm						
*S.* Dublin grown in anaerobic ½ BHI broth	0.80 ^c^	1.04 ^b^	1.04 ^b^	1.38 ^a^	<0.0001	0.052
*S.* Typhimurium grown in anaerobic ½ BHI broth	0.91 ^d^	1.08 ^c^	1.13 ^b^	1. 24 ^a^	<0.0001	0.096
						
Mean specific growth rate (per h)						
*S.* Dublin grown in anaerobic ½ BHI broth	0.95 ^c^	0.90 ^d^	1.07 ^b^	1.118 ^a^	<0.0001	0.014
*S.* Typhimurium grown in anaerobic ½ BHI broth	0.90 ^bc^	0.83 ^c^	1.12 ^a^	1.02 ^ab^	0.0051	0.041
						
Rate of nitrate consumption (µmol nitrate/mL per h)						
*S.* Dublin grown in anaerobic ½ BHI broth	NA	0.90 ^a^	NA	0.68 ^b^	0.0289	0.048
*S.* Typhimurium grown in anaerobic ½ BHI broth	NA	0.71 ^a^	NA	0.26 ^b^	0.0003	0.028
			
Total nitrate consumed by 12 h (µmol nitrite/mL) ^3^						
*S.* Dublin grown in anaerobic ½ BHI broth	NA	3.76	NA	3.67	0.1024	0.032
*S.* Typhimurium grown in anaerobic ½ BHI broth	NA	2.84 ^a^	NA	1.35 ^b^	0.0086	0.219
						
Peak nitrite produced from nitrate (µmol nitrite/mL)						
*S.* Dublin grown in anaerobic ½ BHI broth	NA	3.03	NA	3.38	0.4906	0.330
*S.* Typhimurium grown in anaerobic ½ BHI broth	NA	1.35 ^a^	NA	0.83 ^b^	0.0111	0.082
						
Total nitrite consumed by 12 h (µmol nitrite/mL)						
*S.* Dublin grown in anaerobic ½ BHI broth	NA	1.85 ^a^	NA	0.29 ^b^	0.0189	0.289
*S.* Typhimurium grown in anaerobic ½ BHI broth	NA	2.70 ^a^	NA	0.52 ^b^	0.0006	0.157
						
Rate of ammonia accumulation (µmol/mL per h)						
*S.* Dublin grown in anaerobic ½ BHI broth	0.02 ^b^	0.11 ^a^	0.01 ^b^	0.11 ^a^	<0.0001	0.079
*S.* Typhimurium grown in anaerobic ½ BHI broth	0.02 ^c^	0.08 ^a^	0.03 ^c^	0.05 ^b^	<0.0001	0.046
						
Net ammonia accumulation (µmol/mL)						
*S.* Dublin grown in anaerobic ½ BHI broth	0.18 ^b^	0.90 ^a^	0.13 ^b^	0.87 ^a^	<0.0001	0.063
*S.* Typhimurium grown in anaerobic ½ BHI broth	0.16 ^c^	0.66 ^a^	0.21 ^c^	0.41 ^b^	<0.0001	0.037

^1^ Nitrate concentration, when administered alone or combined with tungstate, at the start of incubation were 3.78 ± 0.32 and 2.85 ± 0.71 µmol/mL for *S*. Dublin and *S*. Typhimurium cultures, respectively. Tungstate was added to achieve 50 mM when administered alone or combined with nitrate. ^2^ Results are from a general analysis of variance with an LSD separation of means. SEM, standard error of the mean. ^3^ Calculated as the difference between amount of nitrate catabolized and residual nitrite measured after 8 h incubation. ^a,b,c,d^ Least square means within rows with unlike superscript differ (*p* < 0.05).

## Data Availability

The authors confirm that the data supporting the findings presented here are included within this article and further inquiries can be directed to the corresponding author.

## References

[1] Dewey-Mattia D., Manikonda K., Hall A.J., Wise M.E., Crowe S.J. (2018). Surveillance for foodborne disease outbreaks—United States, 2009–2015. MMWR Surveill. Summ..

[2] Canning M., Birhane M.G., Dewey-Mattia D., Lawinger H., Cote A., Gieraltowski L., Schwensohn C., Tagg K.A., Francois Watkins L.K., Robyn M.P. (2023). *Salmonella* outbreaks linked to beef, United States, 2012–2019. J. Food Prot..

[3] Pullinger G.D., Paulin S.M., Charleston B., Watson P.R., Bowen A.J., Dziva F., Morgan E., Villarreal-Ramos B., Wallis T.S., Stevens M.P. (2007). Systemic translocation of *Salmonella enterica* serovar Dublin in cattle occurs predominantly via efferent lymphatics in a cell-free niche and requires type III secretion system 1 (T3SS-1) but not T3SS-2. Infect. Immun..

[4] Lopez C.A., Rivera-Chávez F., Byndloss M.X., Bäumler A.J. (2015). The periplasmic nitrate reductase NapABC supports luminal growth of *Salmonella enterica* serovar Typhimurium during colitis. Infect. Immun..

[5] Tiso M., Schechter A.N. (2015). Nitrate reduction to nitrite, nitric oxide and ammonia by gut bacteria under physiological conditions. PLoS ONE.

[6] Vázquez-Torres A., Bäumler A.J. (2016). Nitrate, nitrite and nitric oxide reductases: From the last universal common ancestor to modern bacterial pathogens. Curr. Opin. Microbiol..

[7] Winter S.E., Winter M.G., Xavier M.N., Thiennimitr P., Poon V., Keestra A.M., Laughlin R.C., Gomez G., Wu J., Lawhorn S.D. (2013). Host-derived nitrate boosts growth of *E. coli* in the inflamed gut. Science.

[8] Worley M.J. (2023). *Salmonella* bloodstream infections. Trop. Med. Infect. Dis..

[9] Moreno-Vivián C.P., Cabello P., Martínez-Luque M., Blasco R., Castillo F. (1999). Prokaryotic nitrate reduction: Molecular properties and functional distinction among bacterial nitrate reductases. J. Bacteriol..

[10] Stewart V. (1988). Nitrate respiration in relation to facultative metabolism in enterobacteria. Microbiol. Rev..

[11] Cole J.A., Richardson D.J. (2008). Respiration of nitrate and nitrite. EcoSal Plus.

[12] Sparacino-Watkins C., Stolz J.F., Basu P. (2014). Nitrate and periplasmic nitrate reductases. Chem. Soc. Rev..

[13] Zhong Q., Kobe B., Kappler U. (2020). Molybdenum enzymes and how they support virulence in pathogenic bacteria. Front. Microbiol..

[14] Zhu W., Winter M.G., Byndloss M.X., Spiga L., Duerkop B.A., Hughes E.R., Büttner L., de Lima Romão E., Behrendt C.L., Lopez C.A. (2018). Precision editing of the gut microbiota ameliorates colitis. Nature.

[15] Yang J., Peng P., Tan S., Ge S., Xie L., Zhou T., Liu W., Zhang K., Zhang Z., Liu J. (2023). Calcium tungstate microgel enhances the delivery and colonization of probiotics during colitis via intestinal ecological niche occupancy. ACS Cent. Sci..

[16] Edrington T.S., Schultz C.L., Bischoff K.M., Callaway T.R., Looper M.L., Genovese K.J., Jung Y.S., McReynolds J.L., Anderson R.C., Nisbet D.J. (2004). Antimicrobial resistance and serotype prevalence of *Salmonella* isolated from dairy cattle in the southwestern United States. Microb. Drug Resist..

[17] Leyendecker S.A., Callaway T.R., Anderson R.C., Nisbet D.J. (2004). Technical note on a much simplified method for collecting ruminal fluid using a nylon paint strainer. J. Sci. Food Agric..

[18] Bryant M.P., Burkey L.A. (1953). Cultural methods and some characteristics of some of the more numerous groups of bacteria in the bovine rumen. J. Dairy Sci..

[19] Anderson R.C., Jung Y.S., Oliver C.E., Horrocks S.M., Genovese K.J., Harvey R.B., Callaway T.R., Edrington T.S., Nisbet D.J. (2007). Effects of nitrate or nitro supplementation, with or without added chlorate, on *Salmonella enterica* serovar Typhimurium and *Escherichia coli* in swine feces. J. Food Prot..

[20] Gates A.J., Hughes R.O., Sharp S.R., Millington P.D., Nilavongse A., Cole J.A., Leach E.-R., Jepson B., Richardson D.J., Butler C.S. (2003). Properties of the periplasmic nitrate reductases from *Paracoccus pantotrophus* and *Escherichia coli* after growth in tungsten-supplemented media. FEMS Microbiol. Lett..

[21] Anderson R.C., Rasmussen M.A. (1998). Use of a novel nitrotoxin-metabolizing bacterium to reduce ruminal methane production. Bioresour. Technol..

[22] Bell N.L., Anderson R.C., Callaway T.R., Franco M.O., Sawyer J.E., Wickersham T.A. (2017). Effect of monensin inclusion on intake, digestion, and ruminal fermentation parameters by *Bos taurus indicus* and *Bos taurus taurus* steers consuming bermudagrass hay. J. Anim. Sci..

[23] Chaney A.L., Marbach E.P. (1962). Modified reagents for determination of urea and ammonia. Clin. Chem..

[24] Cataldo D.A., Haroon M., Schrader L.E., Youngs V.L. (1975). Rapid colorimetric determination of nitrate in plant tissue by nitration of salicylic acid. Commun. Soil Sci. Plant Anal..

[25] Schneider N.R., Yeary R.A. (1973). Measurement of nitrite and nitrate in blood. Am. J. Vet. Res..

[26] Goetsch A.L., Galyean M.L. (1985). Influence of feeding frequency on passage of fluid and particulate markers in steers fed a concentrate diet. Can. J. Anim. Sci..

[27] Sarker N.C., Keomanivong F., Borhan M., Rahman S., Swanson K. (2018). In vitro evaluation of nano zinc oxide (nZnO) on mitigation of gaseous emissions. J. Anim. Sci. Technol..

[28] Chalupa W. (1977). Manipulating rumen fermentation. J. Anim. Sci..

[29] Marais J.P., Therion J.J., Mackie R.I., Kistner A., Dennison C. (1988). Effect of nitrate and its reduction products on the growth and activity of the rumen microbial population. Br. J. Nutr..

[30] Wang H., Gunsalus R.P. (2000). The *nrfA* and *nirB* nitrite reductase operons in *Escherichia coli* are expressed differently in response to nitrate than to nitrite. J. Bacteriol..

[31] Kaspar H.F., Tiedje J.M. (1981). Dissimilatory reduction of nitrate and nitrite in the bovine rumen: Nitrous oxide production and effect of acetylene. Appl. Environ. Microbiol..

